# SBMLeditor: effective creation of models in the Systems Biology Markup Language (SBML)

**DOI:** 10.1186/1471-2105-8-79

**Published:** 2007-03-06

**Authors:** Nicolas Rodriguez, Marco Donizelli, Nicolas Le Novère

**Affiliations:** 1 European Bioinformatics Institute, Wellcome Trust Genome Campus, Hinxton, Cambridge, UK

## Abstract

**Background:**

The need to build a tool to facilitate the quick creation and editing of models encoded in the Systems Biology Markup language (SBML) has been growing with the number of users and the increased complexity of the language. SBMLeditor tries to answer this need by providing a very simple, low level editor of SBML files. Users can create and remove all the necessary bits and pieces of SBML in a controlled way, that maintains the validity of the final SBML file.

**Results:**

SBMLeditor is written in JAVA using JCompneur, a library providing interfaces to easily display an XML document as a tree. This decreases dramatically the development time for a new XML editor. The possibility to include custom dialogs for different tags allows a lot of freedom for the editing and validation of the document. In addition to Xerces, SBMLeditor uses libSBML to check the validity and consistency of SBML files. A graphical equation editor allows an easy manipulation of MathML. SBMLeditor can be used as a module of the Systems Biology Workbench.

**Conclusion:**

SBMLeditor contains many improvements compared to a generic XML editor, and allow users to create an SBML model quickly and without syntactic errors.

## Background

### Systems Biology Markup Language – SBML

In Systems Biology, complementary computational tools are often used to model and analyse different characteristics of a particular system. Most tools have their own specific format for entering and storing models, and switching tools not long ago most often required re-writing the model from scratch. Moreover, when a tool was no longer supported, models could be lost forever.

The Systems Biology Markup Language (SBML) is a free, open, XML-based format designed to promote interoperability between different tools [1]. Model descriptions produced by one tool can be read and processed by other programs. It also offers a standard representation for model storage, transmission, and re-use. SBML can be used for describing models of (but not restricted to) signalling pathways, metabolic networks, gene regulation networks, etc. SBML is specified as a set of class descriptions, and can be implemented in very different ways. Currently it is mainly instantiated as an XML language [2].

Although SBML is text-based, and can be edited in a simple text editor, it is intended to be written and read by machines, not by human beings. As such, it requires specific user-interfaces to translate modeller's intentions into its computer representation.

### SBMLeditor

The main target users of this SBML editor are scientists using different software to develop and simulate their models and who spend significant time editing by hand their models, either to enter small modifications or even to write complete models. The need to build a specific tool to facilitate the quick creation and editing of correct SBML files has been growing both with the number of SBML users, and the increased complexity of the SBML Level 2 format [3] (a problem that will increase with the future Level 3). SBMLeditor tries to answer these needs by providing a very simple, low level editor of SBML files. Users can create and remove all the necessary bits and pieces of SBML in a controlled way, that maintains the validity and consistency of the final SBML file. In addition, SBMLeditor provides a tool covering 100% of the SBML specifications, either Level 1 or Level 2, without being hindered by software-specific constraints. Finally, this editor allows investigators to implement and test quickly new SBML proposals, such as the controlled annotations part of SBML Level 2 Version 2 [4].

## Implementation

SBMLeditor is written with the Java GUI toolkit Swing. Xerces [5] is used to parse the SBML files through the Document Object Model (DOM) [6]. JCompneur, a library written by Marco Donizelli, is then used to display and edit this DOM tree as a Java JTree. JCompneur has already been used in several projects to edit file according to differents XML schemas, and is quite easy to use and customize for any need. It offers a serie of Java interfaces that one can use to create, edit and filter XML nodes. The creation and editing of XML nodes is made following the Template pattern [7] with preAction(), action() and postAction() methods. During the action, a customized JPanel is displayed to the user, where he can enter the required information. Wherever it is possible, the choice offered to the user is restricted with lists of choices, reducing the selection errors. However, many constraints on a SBML file are only defined in the SBML specification, and not in the XML schema. Therefore, some errors can go through despite the built-in constraints. We use libSBML [8], the library written by the SBML Team, to check the consistency of the resulting SBML file. This library is written in C, with a Java interface using the Simplified Wrapper and Interface Generator (SWIG) [9].

LibSBML is also used to manipulate the Mathematical Markup Language (MathML) [10] content, included in some of the SBML elements. A function is used to transform the MathML into an infix formula and conversely. All the references to SBML elements in MathML expressions are made to the attribute "id" of the elements. The value of these ids is restricted by a pattern (see section 3.1.7 of the SBML specifications) and some software might create unique ids without meaning. Each relevant SBML element also possess an attribute "name", supposed to be human readable. Each id is substitued by the corresponding name if it exists. The resulting formula is then displayed in a customize Jex [11] panel, where some coloring is added to distinguish between the different types of SBML element.

### Main interface

**Figure 1:**
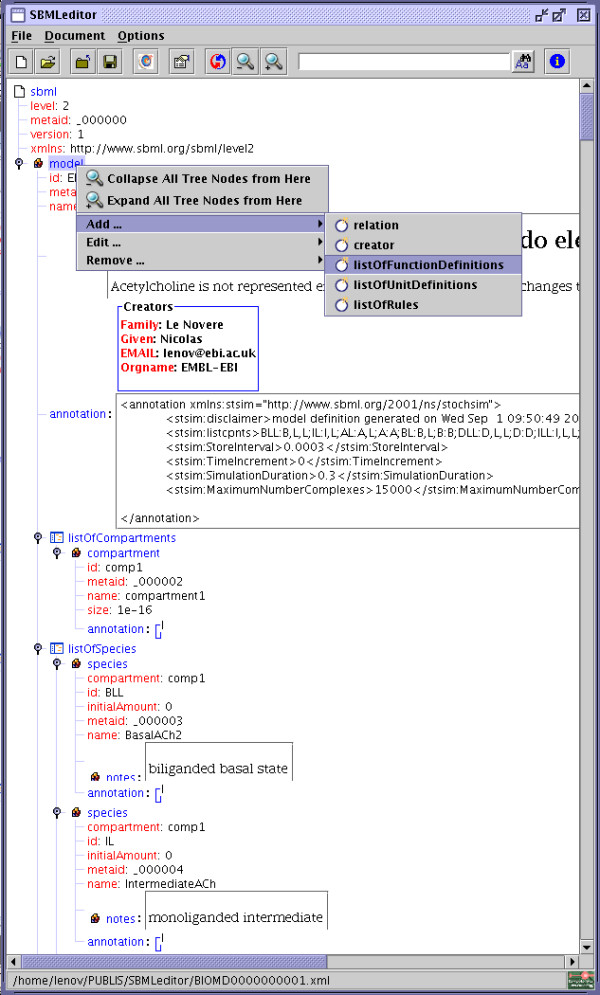
**Main window**. Main frame of the application, showing the general menu, the toolbar, and the context menu associated with the element "model".

Once an SBML file is opened, the structure of the model is represented as a tree (Figure 1). The main menu and a tool bar on top of the window offer access to the generic editing and validation functions. Note that the content of the main menu depends on the context, i.e. of the selected node. Almost all the functions are also available through keyboard shortcuts and/or local context menus, in order to facilitate the navigation inside the file and limit as much as possible the unecessary use of the pointer tracker. For each SBML element, the local contextual menu offers a choice of options such as adding a child, editing the element, removing it etc., plus generic fold/unfold functions.

### Library for XML editing

In order to facilitate the development of several projects involving the editing of XML files, we developed a generic library, JCompneur. SBMLeditor is built on top of the library, with only a limited number of additions. A custom dialog is created to allow one to create or edit SBML elements. The NodeTreeCellRenderer class has been extended to customize the display of the tree. For instance, SBMLeditor does not display the complete DOM tree of the elements *notes, annotation *or *math*, containing XML elements that do not belong to SBML namespace. Instead, the editor displays the sub-tree inside a TextArea. In turn, this allows users to customise the rendering of some subtree in the editor. For instance, the content of the element *notes *is XHTML. In the tree, the XHTML is interpreted and the notes are displayed as they would be in a web browser, the XHTML tags being only visible in the editing window. The methods create and process node are based on the Template Pattern. For example, the create method is splitted into the following steps:

• Invoke preCreate.

• Set some properties (size, location, etc.) of the dialog.

• Make the dialog visible.

• Wait until the dialog is no longer visible.

• If the *OK *button was pressed, invoke postCreate otherwise invoke resetCreate

• Return the newly created XML DOM element.

### Dialogs

**Figure 2:**
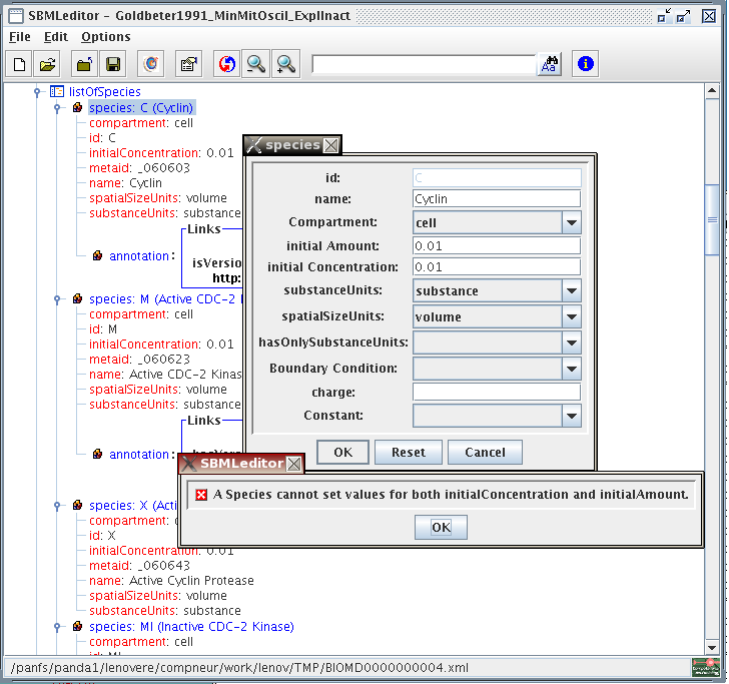
**Editing a Species**. Dialog window allowing the edition of a species. Note the validation rule that does not allow to enter both an initialConcentration and an initial Amount.

The editing of SBML elements is done through dedicated dialog windows. These dialogs provide help and constraints to the user, allowing to create a new SBML document from scratch easily and quickly. A dialog displays all the attributes allowed in the current element. Whenever possible, a list of permitted values has been created, rather than using free text, as in a standard XML editor. The SBML specification contains many constraints relative to the different elements or attributes. Most of these constraints are not described in the SBML schema. Therefore we expressed them directly in the user interface by restraining the possible values, or by validating them before accepting the modifications. Figure 2 shows the configuration of an element *species*. There are only a few numerical fields that are free text, all the other attributes being set-up through a list of possible values. The units fields contain a list of all the units defined in the model, plus all the units predefined in the current SBML specification. In the case of the attribute *compartment*, the list contains all the compartments already defined in the model. As with every widget (except the raw MathML editing) the *name *of the compartment is displayed rather than its *id*. The boolean fields have only three values, true, false and the empty string.

As a consequence of the use of pull-down lists of existing values, a user is forced to build the model hierarchicaly, in the order compartment>species>parameter>reaction etc., minimizing the risk of syntactic errors. An exception is the use of the graphical MathML editor (see below).

### Controlled annotation

All SBML elements can be annotated in a controled manner [4] using the Resource Description Framework (RDF) [12]. The editor can parse each annotation element to see if it contains, at the top level, an RDF element, and that this RDF element complies with the format cited above. Additional annotation is entered through a dialog that displays predefined resources. Those predefined resources are stored in an XML file provided with the distribution (in the main installation folder), and that can be extended by the user. In addition, custom resources can be added directly as free text.

The definition of a resource in the XML file follows the syntax described below:

resource name="Gene Ontology"

uri="http://www.geneontology.org/"

location="http://www.ebi.ac.uk/ego/"

action="http://www.ebi.ac.uk/ego/DisplayGoTerm?selected="

elements="model compartment species reaction event algebraicRule assignmentRule rateRule"

idInputHelper="GO:"

idPattern="GO:\d7"

The only mandatory fields are name, uri and elements. The attribute elements is a list of the different SBML elements with which the resource can be associated. The attribute name is the only information displayed to the user. The uri is a stable string representing the data type, as described in the MIRIAM standard [13]. MIRIAM, standing for *Minimal Information Requested In the Annotation of Models*, is an effort to standardize the minimal metadata so that different groups can collaborate on annotating and curating computational models in biology. On the contrary of the uri, location and action are dependent of the user configuration and could vary from one user to the other. The idInputHelper and idPattern fields help in defining a valid identifier.

### Equation editor

**Figure 3:**
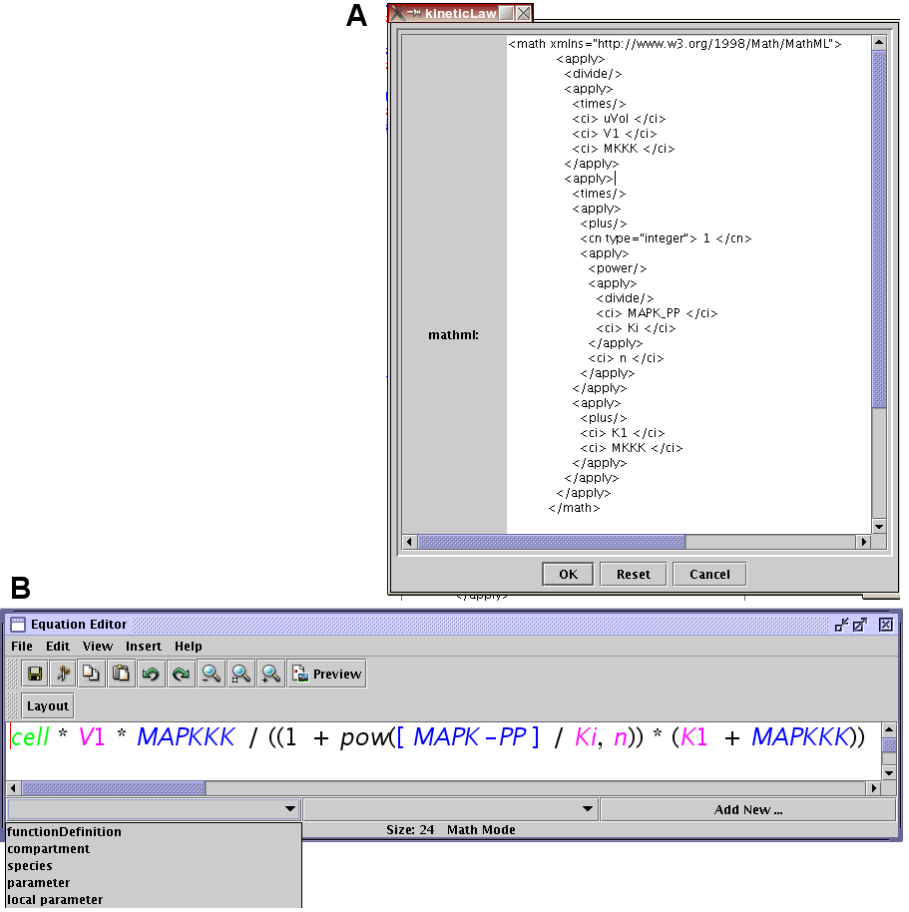
**Editing MathML**. Panel A, direct text-edition of the MathML code; panel B, graphical edition of the mathematicla expressions. The colors represent the namespaces of the symbols. Note that the name of entities are used rather than their identifiers, following SBML guidelines.

One of the main problems of writting SBML Level 2 files by hand is the use of the Mathematical Markup Language (MathML) [10] to describe all the mathematical portions of the model. MathML expressions are long, complex, and therefore error-prone. To facilitate the editing of mathematical expressions, we designed a small equation editor based on jex [11]. The user can type the equation as free text, in a natural infix notation, that is subsequently transformed into valid MathML using the subset of MathML elements supported by SBML (Figure 3). The math editor also provides several drop-down menus (JList) to display the different elements that are already defined in the model and can be included in the equation (again using the *name *instead of the *id *to improve the readability of the equation). There is also the possibility to add new SBML elements *compartments, species *or *parameters *from within the math editor window. This avoids the necessity to go back to the main window if the declaration of an element has been forgotten.

### Consistency checking

A number of rules must be checked to be sure of the complete validity of the SBML model. A web page regrouping all these rules has been created to help software developers in checking all of them [14]. We implemented some of these consistency checks in SBMLeditor, but most of them are performed using the function checkConsistency of libSBML.

### System Biology Workbench (SBW)

**Figure 4:**
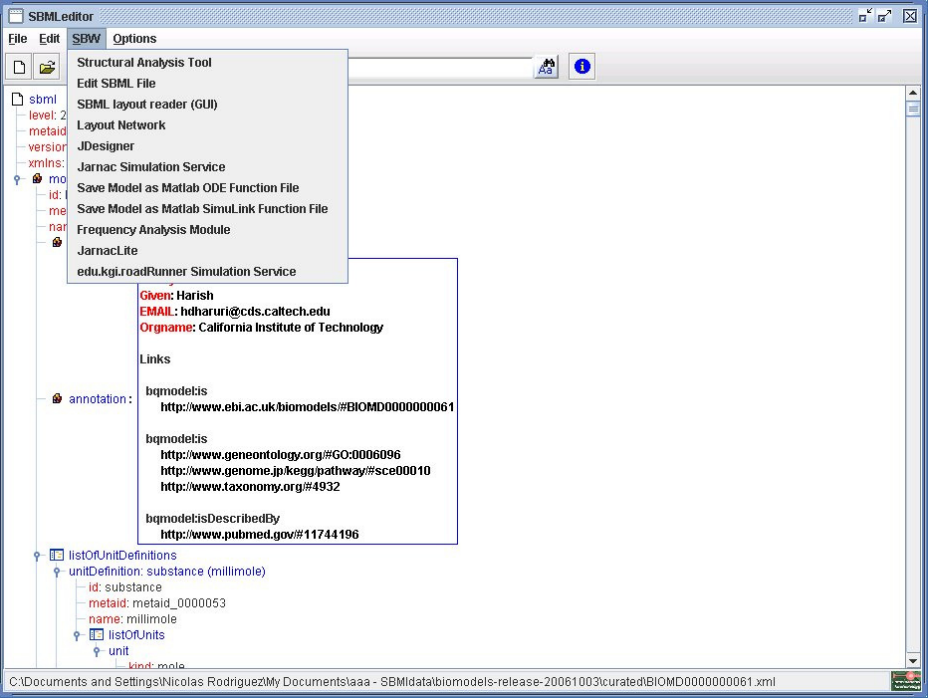
**SBW menu**. Use of the SBW menu under Windows, showing the available modules.

The System Biology Workbench (SBW) is a software framework that allows heterogeneous application components-written in diverse programming languages and running on different platforms-to communicate and use each others' capabilities [15]. The support for SBW has been added to SBMLeditor by the project SIMAP [16]. We can register the SBMLeditor as an SBW module. Any applications capable of talking to SBW can therefore open SBMLeditor through SBW, and edit and modify their SBML files before continuing the modelling and simulation process. Within SBMLeditor, we created a menu that lists all the analysis services registered to SBW (Figure 4). One can then launch Jarnac or JDesigner with the SBML model edited with SBMLeditor.

### Problems and future developments

• The XML parsing using DOM is not efficient for large files. SBMLeditor was not originally conceived to manipulate large models. Since large models already exist and will become more frequent (in particular when model composition will become mainstream), we have to think about some way to help their editing.

• More methods need to be developed to check the consistency of the model. Some will be developed within libSBML, while some will have to be included in the editor itself.

• Support for the forthcoming SBML Level 2 Version 2 has to be completed.

• Contextual help has to be added for each dialog, that will include both the relevant part of the SBML specification and the SBMLeditor specific help.

• Support for the Systems Biology Ontology (SBO) [17] has to be implemented.

## Conclusion

SBMLeditor is a fully functional editor for modellers who want to develop a model *de novo *in SBML, but also for scientists who need to read an SBML model generated by another tool. As an example, SBMLeditor is used by the curators of BioModels Database [18] to encode models, or to curate models submitted to the database by third-parties. SBMLeditor allows users to develop a model more quickly and with less errors than a generic XML editor.

## Availability and requirements

The SBMLeditor is distributed the GNU General Public License (GPL). Distribution for several environments including the code source and some data can be downloaded at .

## Authors' contributions

MD developed a library which provide interfaces to easily display an XML document as a java tree. NR developed the editor itself and NLN wrote the specifications, listed feature requirements and is the main tester.
